# Surface Modification of Matrimid^®^ 5218 Polyimide Membrane with Fluorine-Containing Diamines for Efficient Gas Separation

**DOI:** 10.3390/membranes12030256

**Published:** 2022-02-24

**Authors:** Tae Hoon Lee, Byung Kwan Lee, Jin Sung Park, Jinmo Park, Jun Hyeok Kang, Seung Yeon Yoo, Inho Park, Yo-Han Kim, Ho Bum Park

**Affiliations:** 1 Department of Energy Engineering, Hanyang University, Seoul 04763, Korea; come2lth@hanyang.ac.kr (T.H.L.); wndghkch@hanyang.ac.kr (B.K.L.); jinsung0902@hanyang.ac.kr (J.S.P.); joon927@hanyang.ac.kr (J.H.K.); lori921@hanyang.ac.kr (S.Y.Y.); inhopark@hanyang.ac.kr (I.P.); 2 H_2_ Technology, R&D Division, KOGAS Research Institute, Incheon 21993, Korea; mayer@kogas.or.kr

**Keywords:** Matrimid^®^ 5218, polyimide, membrane, gas separation, diamine modification

## Abstract

Polyimide membranes have been widely investigated in gas separation applications due to their high separation abilities, excellent processability, relatively low cost, and stabilities. Unfortunately, it is extremely challenging to simultaneously achieve both improved gas permeability and selectivity due to the trade-off relationship in common polymer membranes. Diamine modification is a simple strategy to tune the separation performance of polyimide membranes, but an excessive loss in permeability is also generally observed. In the present work, we reported the effects of diamine type (i.e., non-fluorinated and fluorinated) on the physicochemical properties and the corresponding separation performance of a modified membrane using a commercial Matrimid^®^ 5218 polyimide. Detailed spectroscopic, thermal, and surface analyses reveal that the bulky fluorine groups are responsible for the balanced chain packing modes in the resulting Matrimid membranes compared to the non-fluorinated diamines. Consequently, the modified Matrimid membranes using fluorinated diamines exhibit both higher gas permeability and selectivity than those of pristine Matrimid, making them especially effective for improving the separation performance towards H_2_/CH_4_ and CO_2_/CH_4_ pairs. The results indicate that the use of fluorinated modifiers may offer new opportunities to tune the gas transport properties of polyimide membranes.

## 1. Introduction

Membrane-based gas separation has drawn significant interest as an energy-efficient process to supplement or alternate the conventional thermally-driven separation methods such as distillation and thermal/pressure-swing adsorption [1,2]. Typical applications of gas separation membranes include hydrogen separation from a purge gas during ammonia separation (H_2_/N_2_), light hydrocarbon reforming (H_2_/CH_4_), capturing greenhouse gases from a coal-fired power plant (CO_2_/N_2_), and natural gas purification (CO_2_/CH_4_) [2,3], which are expected to serve as essential technologies towards carbon neutrality to resolve global climate change [4,5,6].

Currently, polymers are the most frequently investigated membrane materials due to their good processability, cost-effectiveness, and excellent mechanical robustness for modulization [7,8,9]. Among a myriad of polymer candidates, such as polymers adopted in commercialized membrane systems (e.g., polysulfone, polyethersulfone, cellulose acetate, and silicon rubber) [10], polyimide has been of interest for decades [11]. This is because aromatic polyimides generally exhibit high diffusivity selectivity, desirable physical properties, and outstanding thermal/chemical stabilities, which are mainly attributed to their rigid backbone structure [12,13,14]. Hence, many researchers have focused on the design and synthesis of a variety of polyimides by coupling diverse dianhydrides and diamines [15]. However, it is extremely difficult to obtain simultaneous enhancements in both permeability and selectivity for polyimides, as this is limited by the well-known trade-off relationship observed in most polymer membranes. Moreover, the custom synthesis of new polyimides is very laborious and time-consuming and thus may not be practical for large-scale applications [11].

In this regard, post-modification of existing polyimides has been explored as a simple and efficient strategy. Common post-treatment protocols can be classified into blending (e.g., polymer blends and mixed matrix membranes) [16,17,18,19], physical modification (e.g., surface coating, annealing, and ultraviolet (UV) beam irradiation) [20,21,22,23], and chemical modification (e.g., functionalization, grafting, and crosslinking) [2,11,15]. For polyimides, chemical modification is regarded as the most promising post-treatment process since it takes advantage of its well-known chemistry, simple processing, and corresponding desirable membrane performance such as improved selectivity as well as plasticization resistance [3,11,24,25].

Diamine modification of polyimides can be performed at room temperature by simply immersing the membrane coupon into a solution containing diamine and swelling solvent, making this method a common way to control the gas separation abilities of polyimide membranes. For example, Tin et al. used *p*-xylenediamine to modify a commercial polyimide membrane. Compared to the pristine membrane, they significantly enhanced He/N_2_ selectivity from 10 to 86 and O_2_/N_2_ selectivity from 4.1 to 5.9 [26]. Unfortunately, an excessive loss in permeability also occurred in the modified membranes (21% loss in He permeability and 47% loss in O_2_ permeability, respectively). These results are generally observed in most diamine-modified polyimides because of their crosslinked structures caused by the formation of amide bonds, which restrict chain mobility [11,27,28,29,30,31]. Although many diamine modifiers have been proposed and utilized to modify polyimide membranes, their separation performances are still seemingly limited by the trade-off relationship [11,31].

From the structural aspects of polyimides, it was recognized that the substitution of hydrogen to fluorine atoms in the polymer chain significantly influences the properties of gas separation [32]. There are huge size differences between hydrogen and fluorine atoms, as indicated by their covalent radii (0.3 Å vs. 0.64 Å) and Van der Waals radii (1.10 Å vs. 1.35 Å), which are responsible for the relatively longer C–F bond compared to the C–H bond [33]. Thus, the bulky fluorine groups are more prone to disrupting chain packing in polymers compared to their hydrogen counterparts [34]. In addition, the highly polar C–F bonds may boost the dipole–dipole interactions between the gas molecules and the polymer chains, which affects the solubility selectivity of the membranes [32,34,35]. Smith et al. investigated the effects of aliphatic and aromatic fluorine groups in 4,4′-(hexafluoroisopropylidene)diphthalic anhydride (6FDA)-based polyimides [35]. It was revealed that the fluorinated analogs exhibit higher gas permeability than their hydrocarbon-based control groups, as governed by the accelerated diffusion of gas molecules through the greater extent of void space induced by bulky fluorine groups.

Considering all these issues, we first report the role of fluorinated diamines in the modified polyimide membranes with a focus on the combined effects of bulky fluorine moiety and diamine-induced crosslinking. Matrimid^®^ 5218 polyimide, consisting of 3,3′-4,4′-benzophenonetetracarboxylic dianhydride (BTDA) and diamino phenylindane (DAPI), was chosen as a representative polyimide precursor due to its commercial availability, well-known physicochemical properties, and high size-sieving abilities [36]. Spectroscopic and thermal characterizations confirmed the successful modification of Matrimid membranes using fluorinated diamines, which are more likely to react at the membrane surface. Interestingly, the fluorinated diamine-modified Matrimid membranes exhibited improvements in both permeability and selectivity, which is in striking contrast to their non-fluorinated diamine counterparts. The structural properties were examined in detail to elucidate the unique gas transport properties of modified membranes using fluorinated diamines.

## 2. Materials and Methods

### 2.1. Materials

**Figure 1:**
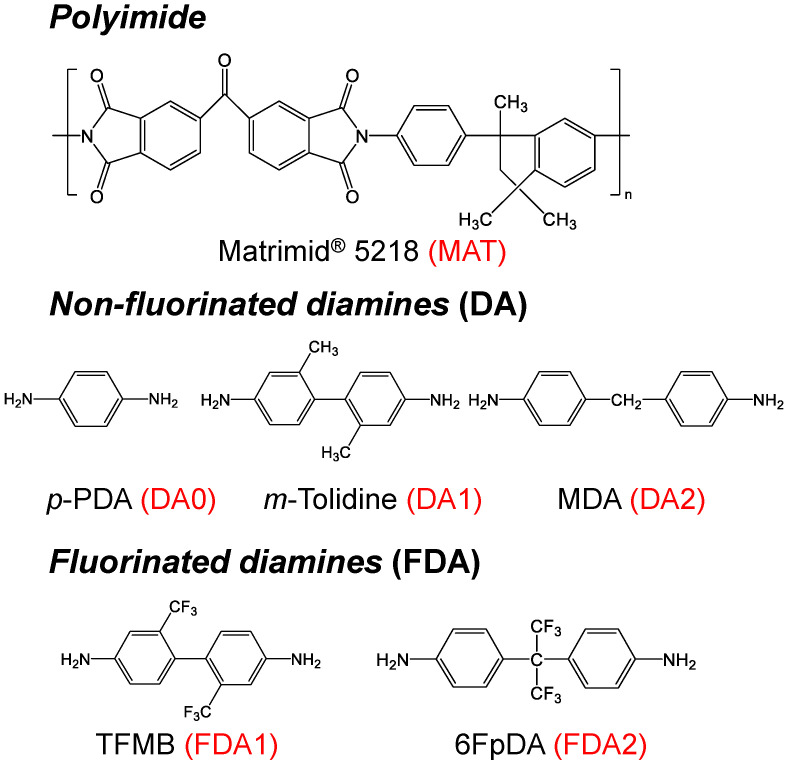
Chemical structures of polyimide and diamines used in this study.

Matrimid^®^ 5218 (Matrimid) was purchased from Alfa Aesar (Haverhill, MA, USA). *p*-Phenylenediamine (*p*-PDA, >98%) and 4,4′-diaminodiphenylmethane (MDA, >97%) were purchased from Sigma Aldrich (St. Louis, MO, USA). *m*-Tolidine (>98%), bis(trifluoromethyl)benzidine (TFMB, >98%), and 2,2-bis(4-aminophenyl)hexafluoropropane (6FpDA, >98%) were purchased from Tokyo Chemical Industry (TCI, Tokyo, Japan). Methanol (MeOH, 99.5%) and N-methyl-2-pyrrolidone (NMP, 99.5%) were purchased from Daejung Chemicals and Metals (Siheung-si, Gyeonggi-do, Korea). All gases for permeation tests were purchased from AirKorea (Yeoju-si, Gyeonggi-do, Korea). Figure 1 illustrates the chemical structures and assigned names of the polyimide (MAT), non-fluorinated diamines (DA), and fluorinated diamines (FDA) used in this study.

### 2.2. Fabrication of Matrimid Membrane

First, 1.4 g of Matrimid powder was completely dissolved in 8.6 g of NMP with vigorous stirring at 120 °C for 12 h. After cooling down to room temperature, the polymer solution was degassed for 20 min using a bath sonicator (CPX8800H-E, Branson, MO, USA) and was subsequently cast onto a glass plate. A dense film was formed by sequential solvent evaporation in a vacuum oven at 80 °C/1 h, 120 °C/1 h, 150 °C/1 h, and 180 °C/8 h. The dried film was peeled off from the plate by immersing it into a water bath and drying it again in a vacuum oven at 120 °C for 24 h. Typically, the resulting dense Matrimid membranes exhibited thicknesses of 50 ± 10 μm.

### 2.3. Diamine Modification of Matrimid Membrane

A 2 × 2 cm^2^-sized coupon of Matrimid membrane was immersed in a MeOH solution containing 10 wt.% of a chosen diamine component at 25 °C for 24 h. Then, the modified film was washed by immersion in MeOH several times to remove residual unreacted diamines and was dried in a vacuum oven at 90 °C for 12 h.

### 2.4. Characterization

Fourier transform infrared (FT-IR) spectra of modified membranes were obtained using a Nicolet 6700 (Thermo electron scientific instruments, Waltham, MA, USA) based on attenuated total reflection (ATR) mode to confirm the incorporation of diamines after modification. The elemental compositions of modified membranes were examined using an X-ray photoelectron spectroscopy analyzer (XPS, K-alpha+, Thermo Fisher Scientific, Waltham, MA, USA) with Al-Kα radiation as the X-ray source. The thermal stability of modified membranes was evaluated using a thermal gravimetric analyzer (Q500, TA Instruments, New Castle, DE, USA) under a nitrogen atmosphere at a heating rate of 10 °C/min. The chain packing mode in a membrane was analyzed using X-ray diffraction (XRD) instrument (Miniflex 600, Rigaku, Tokyo, Japan) with focused monochromatized Cu Kα radiation (λ = 1.5406 Å) at a scan rate of 10 °/min. The glass transition temperature (T_g_) of the membranes was determined using a differential scanning calorimetry (DSC) instrument (Q20, TA Instruments). The sample was first heated to 400 °C and was then cooled to 25 °C at a scan rate of 10 °C/min under a nitrogen atmosphere, and the same procedure was repeated once more. The second cycle was analyzed using Universal Analysis V4.35A (TA instruments) to measure the T_g_ of the membranes. The density of a membrane was obtained by the buoyancy method using hexadecane as the auxiliary liquid. The density of all membranes was calculated as follows:

$$\rho =\rho_{0}\frac{M_{A}}{M_{A}-M_{L}}$$

where ρ (g/cm^3^) and $\rho_{0}$ are the densities of the membrane and hexadecane (0.773 g/cm^3^), respectively, and $M_{A}$ and $M_{L}$ are the weights of the membrane measured in air and liquid, respectively.

### 2.5. Gas Permeation Measurement

The gas transport properties of the modified membranes were evaluated using the constant-volume/variable-pressure method (high vacuum time-lag). Before each test, both the feed side and permeate side were evacuated to less than 10^−6^ Torr using a high vacuum pump. Subsequently, the feed gas was injected into a sample chamber at a target pressure of 35 °C. The continuous increase in pressure at the permeate side was recorded using a pressure transducer (Baraton 626B, MKS Instruments, Andover, MA, USA). The permeability coefficient (P, 1 Barrer = 10^−10^ cm^3^ (STP) cm cm^−2^ s^−1^ cmHg^−1^) was calculated from the time-dependent, linear pressure increase at steady-state as:

$$P=\frac{VT_{0}l}{p_{0}T\Delta pA}\left(\frac{dp}{dt}\right)$$

Here, V (cm^3^) is the volume of the permeate side, l (cm) is the membrane thickness, Δp (cmHg) is the pressure difference between the feed and permeate side, A (cm^2^) is the effective membrane area, T (K) is the measurement temperature, $T_{0}$ and $p_{0}$ are the standard temperature and pressure, respectively, and (dp/dt) is the rate of pressure increase at a steady state. The selectivity was calculated as the ratio of the permeability coefficient of the two gases:

$$Selectivity=\frac{P_{A}}{P_{B}}$$

## 3. Results and Discussion

### 3.1. Preparation and Characterization of Diamine-Modified Matrimid Membranes

**Figure 2:**
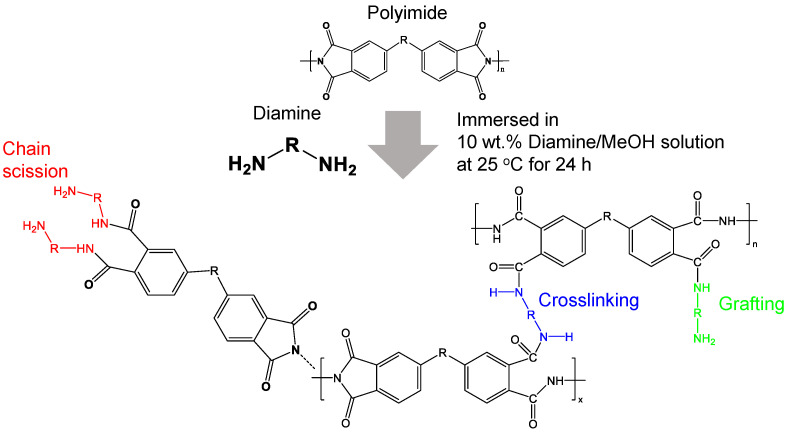
Possible reaction mechanisms of diamine modification of polyimide membranes.

Figure 2 represents possible reaction mechanisms between polyimide chains and diamine molecules [37]. The imide ring on the backbone of the polyimide is easily opened by contact with diamines because of their strong nucleophilicity toward carbonyl groups [11]. Diamine grafting happens when one of the amine groups in the diamine reacts with the imide ring, leaving two amide groups. If two amine groups in the diamine react with two imide rings, crosslinking occurs, and one imide ring is converted into two amide groups [30]. Since the resulting amide groups are susceptible to nucleophilic attacks, sometimes one imide group reacts with two diamine molecules, and thus chain scission occurs in the presence of an excessive concentration of diamines [11,31,37].

Typically, the diamine-based polyimide modification is performed by immersing the polyimide film into an alcoholic diamine solution [11,26,29]. Methanol is a common solvent to effectively swell the polyimide precursor, which allows more accessible polymer chains to increase the reactions with the diamines [31]. The reactivity of polyimide and diamines, diamine concentration, membrane thickness, temperature, and reaction time are important factors that determine the overall reaction rate as well as the degree of crosslinking. In order to investigate the effects of diamine structures in modified Matrimid membranes, we fixed variables such as diamine concentration (10 wt.%), membrane thickness (~50 μm), temperature (25 °C), and reaction time (24 h) to values that were widely adopted in most of the diamine modification studies [26,29,30,31,37,38,39].

**Figure 3:**
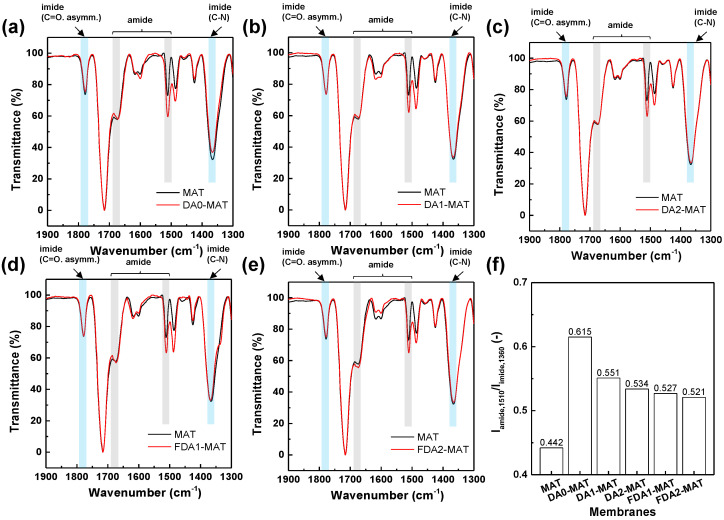
FT-IR analysis of diamine-modified Matrimid membranes fabricated using (**a**) DA0, (**b**) DA1, (**c**) DA2, (**d**) FDA1, and (**e**) FDA2. (**f**) The intensity ratio of amide peak at 1510 cm^−1^ to imide peak at 1360 cm^−1^.

Figure 3a–e shows the Fourier transform infrared (FT-IR) spectra of modified Matrimid membranes depending on the type of diamines, which were normalized from the original data on the basis of the highest FT-IR peak in each spectrum for a better comparison. Here, we designated the modified membranes as OOO-MAT, where MAT stands for Matrimid and OOO stands for non-fluorinated diamine (DA0, DA1, and DA2) or fluorinated diamines (FDA1 and FDA2) used in the reaction, respectively. For all membranes, a slight decrease in the peaks of the imide group (asymmetric C=O stretching at 1775 cm^−1^ and C–N stretching at 1360 cm^−1^) was observed while the peaks of the amide group (C=O stretching at 1672 cm^−1^ and N–H bend at 1510 cm^−1^) became higher or sharper [37,38]. The results indicate that the diamines were successfully incorporated into the polyimide matrix by reaction mechanisms, as described in Figure 2.

The extent of peak intensification varies with diamine type. In order to quantify the degree of reaction, we focused on the amide peak at 1510 cm^−1^ and the imide peak at 1360 cm^−1^ that displayed the most noticeable changes after diamine modification. The intensity ratio of these two peaks (I_amide,1510_/I_imide,1360_) was calculated for each membrane [27]. As shown in Figure 3f, the relative portion of amide was increased in the order of FDA2 < FDA1 < DA2 < DA1 < DA0, indicating that the smallest DA0 diamine underwent the modification reaction most effectively. When comparing the diamine pairs sharing the same molecular structures with or without fluorine-substituted groups (i.e., DA1 vs. FDA1 and DA2 vs. FDA2), the fluorinated diamines exhibited a less pronounced reactivity than that of non-fluorinated ones. Given that the reaction occurs in the solid-state environment, the diffusion of diamines across the polyimide film is expected to be sterically constrained, which increases as the steric hindrance increases as the reaction occurs [31,38,39]. Thus, we speculated that bulkier fluorinated diamines are more likely to react at the membrane surface due to their difficulties permeating through the polymer chains, which causes limited reactivity even though the existence of methanol assists the diffusion of diamines by swelling the membrane at the initial stage.

Note that the sizes of aromatic diamines used in this study are relatively larger than the aliphatic diamines reported in the common literature. For example, Shao et al. used 1,3-propanediamine (PDA) to crosslink the Matrimid membrane, leading to the nearly complete conversion of a polyimide ring into amide groups due to the fast reaction kinetics. They reported that the PDA-modified Matrimid membrane showed an excellent solvent tolerance (i.e., high gel fraction close to 100%) due to the highly crosslinked structure [31]. In contrast, in the present work, we found that all diamine-modified membranes were completely dissolved in the organic solvent (NMP), suggesting that a surface-concentrated reaction occurred when the aromatic diamines reacted with the Matrimid membrane. These results are consistent with the recent report by Kim et al. that investigated the crosslinking of a Matrimid membrane by *p*-phenylenediamine (*p*-PDA, equal to DA0 in this work) by comparing liquid-phase and methanol-swelling-induced post-treatment protocols [38]. They concluded that the Matrimid membrane modified by methanol-swelling protocol (i.e., the same method used in this work) exhibited poor solvent resistance due to the surface-limited reaction on the membrane surface compared with the liquid-phase protocol that induced crosslinking of the entire membrane, including the inner side. Nevertheless, the surface-modified Matrimid membranes have great potential to provide both better thermal and mechanical stability (considering the presence of the rigid and stable imide groups) and improved gas separation performances, which is demonstrated in later sections [37].

In order to further prove the presence of bulky fluorinated diamines on the surface of the resulting Matrimid film, the elemental composition of each membrane was explored by X-ray photoelectron spectroscopy (XPS) given its excellent surface sensitivity (<10 nm depth) [40]. Fluorine contents were detected in FDA-modified Matrimid membranes while they were not observed in the pristine membrane (Table 1). Moreover, FDA2-MAT showed a lower amount of fluorine than that in FDA1-MAT, which is potentially ascribed to the lower reactivity of FDA2 and is consistent with FT-IR analyses.

**Figure 4:**
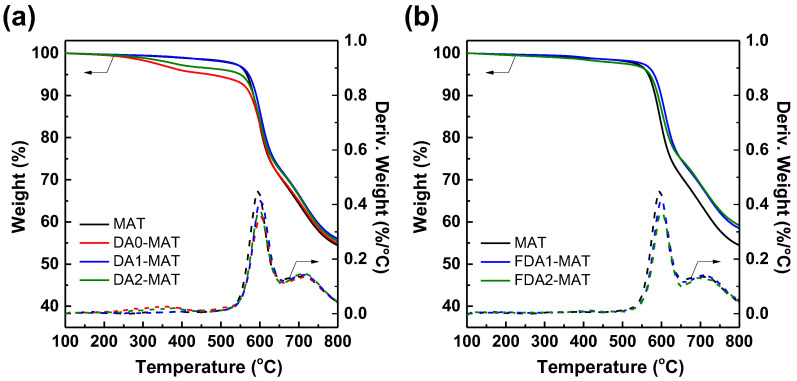
TGA curves of modified Matrimid membranes using (**a**) non-fluorinated diamines and (**b**) fluorinated diamines.

The thermal stability of the modified Matrimid membranes was examined by thermogravimetric analysis (TGA) at a heating rate of 10 °C/min in a nitrogen atmosphere. All membranes exhibited similar thermal decomposition behaviors with some variations (Figure 4). At the initial stage, ~5% weight loss up to 250 °C was attributed to the loss of the residual solvent (NMP) despite vacuum storage after film formation [37]. Although the presence of NMP may influence the gas transport properties of the membranes [41], the same extent of NMP concentration for all membranes allows us to exclude the potential influences of residual solvent safely. For modified membranes, a second area of the decomposition was observed at around 300–400 °C, which indicates the removal of the incorporated amide groups via thermal re-imidization [30]. Lastly, a drastic weight loss occurred above 420 °C due to the thermal decomposition of the imide group and carbonization. Overall, the thermal stability of modified Matrimid membranes in this work is comparable with the bulky 1,3-cyclohexanebis(methylamine) (CHBA)-modified 6FDA-durene membranes [29], whereas the crosslinked Matrimid film fabricated using a relatively smaller ethylenediamine (EDA) was shown to be unstable even at 100 °C [30,31]. Indeed, the results imply that the modification of the Matrimid membrane based on the bulky diamines may be confined to the surface and did not occur throughout the entire membrane.

For non-fluorinated diamines, DA0-MAT showed the most noticeable weight loss, possibly due to having the highest reactivity, which helped it remain in the modified film (Figure 4a). On the other hand, the FDA1-MAT and FDA2-MAT showed less thermal decomposition in the 300–400 °C regime while maintaining a higher residual mass at or above 420 °C compared to that of control groups such as DA1-MAT, DA2-MAT, and the pristine membrane (MAT) (Figure 4b). It is expected that the high strength of the C–F bond is responsible for the better thermal stability in FDA-modified Matrimid membranes because the trifluoromethyl group will decompose when the temperature is sufficiently high [42,43]. Likewise, Pinnau et al. reported that fluorinated aromatic polyimides release the fluorine from a fluorinated diamine group (2,2-bis(trifluoromethyl)benzidine, equal to FDA1) above 600 °C [44].

### 3.2. Gas Separation Performance of Diamine-Modified Matrimid Membranes

**Figure 5:**
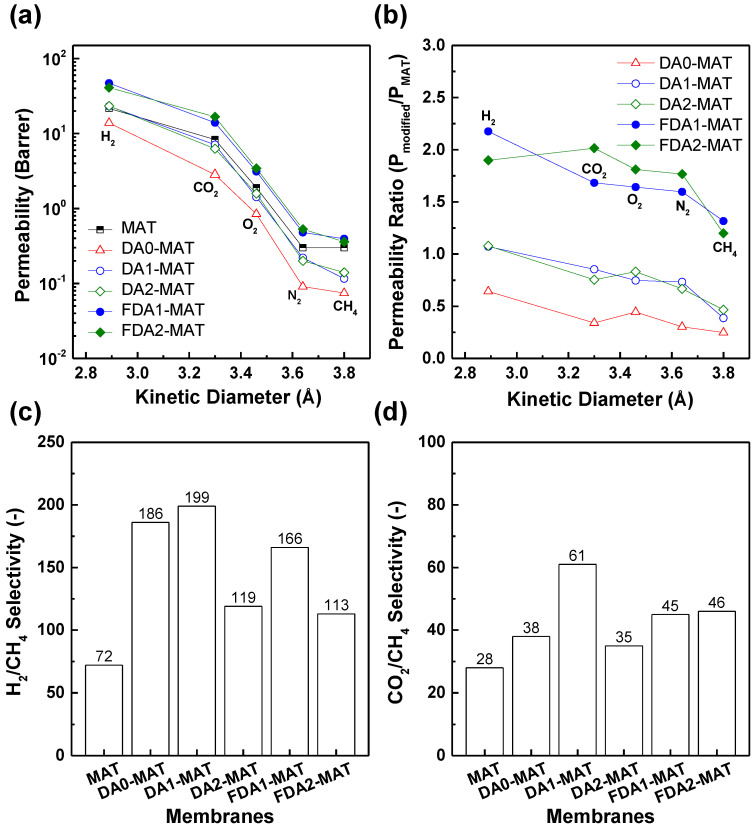
(**a**) Gas permeability of diamine-modified Matrimid membranes depending on the kinetic diameters of gas molecules at 2 bar and 35 °C. (**b**) The corresponding permeability ratios of diamine-modified Matrimid membranes to pristine ones. (**c**) H_2_/CH_4_ selectivity and (**d**) CO_2_/CH_4_ selectivity of diamine-modified Matrimid membranes.

Table 2 and Figure 5a summarize the gas transport properties of diamine-modified Matrimid membranes. Obviously, the gas permeabilities decrease as the kinetic diameter of gas molecules increases (i.e., CH_4_ (3.80 Å) < N_2_ (3.64 Å) < O_2_ (3.46 Å) < CO_2_ (3.30 Å) < H_2_ (2.89 Å)). This is attributed to the low free volume caused by the rigid and densely packed polymer chains in the Matrimid film, which results in the high diffusivity selectivity of Matrimid [36]. In contrast, high free volume polymers (e.g., 6FDA-based polyimides and polymers of intrinsic microporosity (PIMs)) typically possess a moderate diffusivity selectivity and sometimes exhibit a reversal of the size-dependent gas permeability trend (especially for the H_2_/CO_2_ pair) by showing a selectivity close to unity or even less because of the high condensability and preferred sorption of CO_2_ [2,45].

In order to compare the effects of diamine modifier in gas separation performance better, the ratio of gas permeability of modified membranes to that of pristine Matrimid (P_modified_/P_MAT_) was plotted along with the sizes of the gas molecules (Figure 5b). For all membranes, a gradual decrease in permeability ratio occurred with increasing penetrant size. These behaviors were observed in polyimide membranes modified with aliphatic diamines and the accompanying rapid crosslinking reaction. The highly crosslinked structure is known to reduce the free volume size by tightening the polymer chains and thus hinders the transport of large gas molecules (such as CH_4_) while enhancing the diffusivity selectivity towards lighter gases such as H_2_ and CO_2_ [18,26,31,37]. Accordingly, the modified Matrimid membranes showed a remarkable increase in selectivity, while the degree of enhancement differs depending on the gas pair or the type of diamine modifier. When comparing the selectivity toward a gas species of interest against two different-sized gas molecules (e.g., H_2_/N_2_ (Δd (size difference) = 0.75 Å) vs. H_2_/CH_4_ (Δd = 0.91 Å) or CO_2_/N_2_ (Δd = 0.34 Å) vs. CO_2_/CH_4_ (Δd = 0.50 Å)), the increase in the selectivity is more noticeable for gas pairs with larger differences in kinetic diameter (i.e., H_2_/CH_4_ and CO_2_/CH_4_) (Table 2 and Figure 5c,d). These results suggest that all modified membranes should undergo the crosslinking reaction by the inclusion of the diamines to improve the separation abilities.

Notably, the permeability ratio also varies significantly depending on the type of applied diamine modifier. Non-fluorinated diamines led to a large reduction in gas permeability in the corresponding modified Matrimid membrane compared with the pristine one (except for H_2_ in the case of DA1-MAT and DA2-MAT) while achieving a substantial increase in selectivity. The extent of permeability reduction follows the order of DA2-MAT ≈ DA1-MAT < DA0-MAT, possibly because of the higher crosslinking degree of DA0-MAT, considering that the smallest diamine results in reaction throughout the membrane. These are consistent with the previous characterization results. On the other hand, the fluorinated diamines-modified Matrimid membranes exhibited a remarkable increase in gas permeability (more than a two-fold increase in H_2_ permeability) coupled with a moderate increase in selectivity. Such a simultaneous improvement in both permeability and selectivity is highly desirable but unusual because of the well-known trade-off relationship in polymeric membranes. These unique gas transport behaviors suggest that the structural properties of FDA1-MAT and FDA2-MAT may differ from those of DA1-MAT and DA2-MAT, for which the diamine modifiers are, respectively, identical except for the existence of fluorine groups.

**Figure 6:**
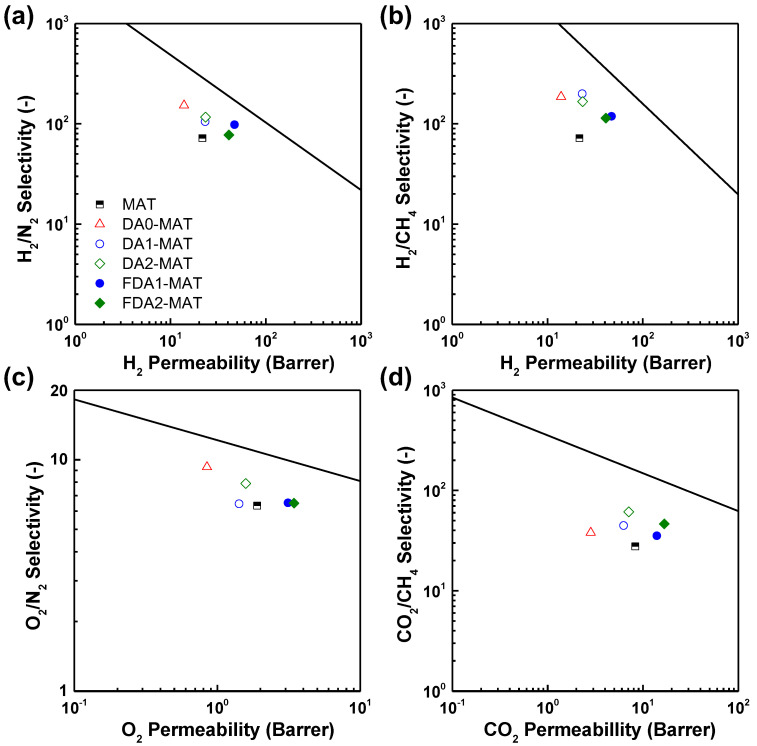
Gas separation performance of diamine-modified Matrimid membranes for (**a**) H_2_/N_2_, (**b**) H_2_/CH_4_, (**c**) O_2_/N_2_, and (**d**) CO_2_/CH_4_ gas pairs measured at 2 bar and 35 °C. Black solid lines represent the 2008 Robeson upper bound for polymer membranes [46].

Figure 6 shows the gas transport data of the diamine-modified Matrimid membranes in comparison with the 2008 Robeson upper bound for pure polymer membranes [46]. It is evident that the post-modification resulted in different trends in gas separation performance of the resulting Matrimid membranes, which are governed by the different gas pairs and the structure of diamine modifiers. For DA0-MAT, the most prominent increase in selectivity was obtained along with the most severe permeability loss. The overall performances of the DA0-MAT membrane are seemingly constrained by the upper bound following the slope of the line. Such a permeability loss was mitigated in modified Matrimid membranes using bulkier non-fluorinated diamines (i.e., DA1-MAT and DA2-MAT), showing upward movement from the original data toward the upper bound line. Most importantly, the gas separation performances (except for similarly sized O_2_/N_2_ pair) of fluorinated diamine-modified membranes approach the upper bound in an inverse way to its slope due to the simultaneous increase in both permeability and selectivity. These results are unprecedented given that previous reports on diamine-modified polyimide membranes generally observed an increase in permeability but reduced permeability (Table 3), which was simply attributed to the membrane densification by crosslinking effects [24,25,29,31,47]. Compared to the pristine Matrimid membrane, FDA2-MAT presents excellent potential in H_2_/CH_4_ and CO_2_/CH_4_ separation applications, which include the production of H_2_ as a renewable energy source for the hydrogen economy, natural gas sweetening, and biogas upgrading [2,3,48,49].

### 3.3. Explaining the Gas Transport Properties of Diamine-Modified Matrimid Membranes: The Role of Fluorine

**Figure 7:**
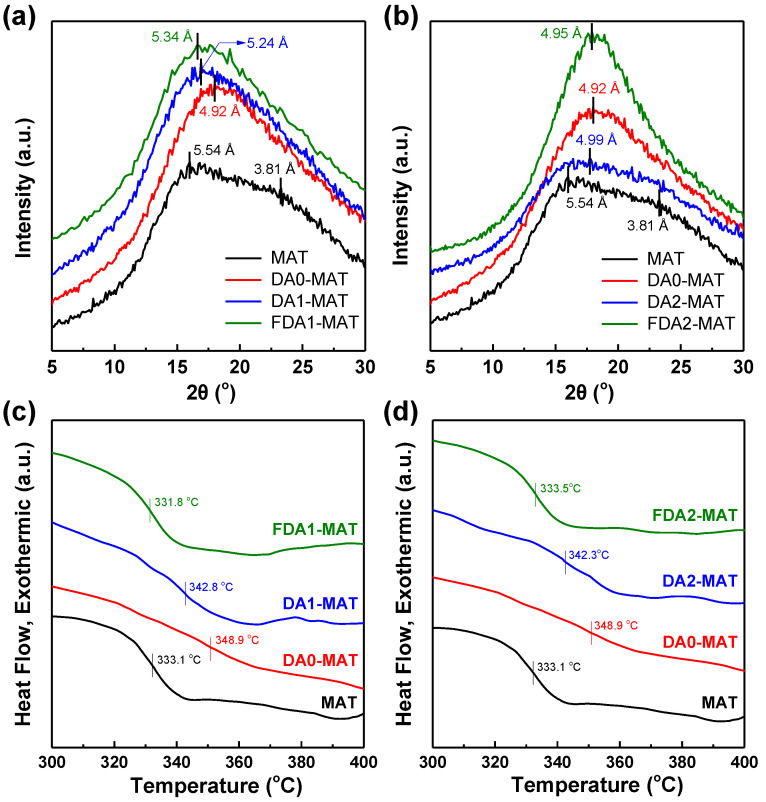
(**a,b**) XRD patterns and (**c**,**d**) DSC curves of diamine-modified Matrimid membranes.

The structural properties of diamine-modified Matrimid membranes were investigated to understand the observed gas separation performances. Evaluation of d-spacing offers useful information on chain packing modes in polymer membranes, which can be obtained by analyzing the X-ray diffraction (XRD) patterns [16,37,50,51], represented in Figure 7a,b. The XRD pattern of the pristine Matrimid film shows two amorphous peaks at 2θ = 16.01° and 23.27°, which are assigned to the average distance between the closely packed chains (d-spacing = 5.54 Å) and the π–π stacking of the aromatic rings (3.81 Å), respectively [52,53]. Notably, the π–π stacking peak is nearly absent in all modified membranes, indicating incorporation of the diamine modifier has induced considerable structural rearrangement in the resulting film.

In Figure 7a, the d-spacing of each membrane corresponding to the interchain distance is found to follow the order of MAT > FDA1-MAT > DA1-MAT > DA0-MAT. The reduced d-spacing after the modification may result from the tightened free volume elements due to the crosslinking reaction with diamines, which is responsible for the improved selectivity of the modified membranes (Table 2) [38,54]. The lowest d-spacing of DA0-MAT can be explained by the fact that DA0 has the highest reactivity [31]. On the other hand, FDA1-MAT showed a higher d-spacing than that of DA1-MAT despite DA1 and FDA1 having the same molecular structure except for the fluorine-substituted groups. Such differences can be attributed to the larger size of fluorine compared with hydrogen, leading to a larger free volume size with less efficient chain packing, resulting in enhanced permeability (Table 2) [32,34,35]. Moreover, the density of the membranes presents an inverted order from the XRD analyses (i.e., MAT < FDA1-MAT < DA1-MAT < DA0-MAT) (Table 4), confirming the d-spacing data since a membrane consisting of a more densely packed structure normally exhibits a higher film density [3,7].

The chain mobility of the membranes can be examined by measuring the glass transition temperature (T_g_) using differential scanning calorimetry (DSC). In general, a higher T_g_ indicates lower chain mobility in a polymer membrane, which can be influenced by diverse factors such as chain-to-chain interactions, backbone rigidity, and steric effects [35]. The T_g_ of the selected membranes follows the order of FDA1-MAT < MAT < DA1-MAT < DA0-MAT (Figure 7c). These results can be ascribed to the competitive effects of the crosslinking-induced interchain forces and the steric hindrance, which depends on the type of diamine modifier. Both DA0-MAT and DA1-MAT exhibited a significant increase in Tg compared to that of pristine Matrimid because the crosslinking may constrict the chain rearrangement [55,56]. DA0-MAT has the highest T_g_, potentially resulting from its highly efficient chain packing, as confirmed by DA0-MAT having the lowest d-spacing. Conversely, FDA1-MAT showed a lower T_g_ than before the modification. We believe that the steric hindrance for chain mobility was sufficiently mitigated in FDA1-MAT due to the more pronounced disruption of chain packing due to its bulky fluorine moieties, whose effects may overcome the mobility limitations by interchain crosslinking [34,35].

On the other hand, a different trend was observed in another pair of modified Matrimid polymers (DA2-MAT and FDA2-MAT) that share a relatively bulkier diamine modifier structure compared to the DA1-MAT and FDA1-MAT cases. Here, the d-spacing of FDA2-MAT was slightly lower than that of DA2-MAT despite the existence of fluorine groups in FDA2 (Figure 7b). This behavior might be attributed to the more accelerated chain rearrangement in FDA2-MAT arising from the largest void space as corroborated by its having the lowest film density compared to others, including the pristine Matrimid (Table 4) [35]. In addition, the amorphous XRD peak of FDA2-MAT is seemingly intensified compared to other modified membranes. Chung et al. investigated the effects of azido-containing monomers in rigid polyimide membranes. From XRD analyses, they observed that the azido incorporation resulted in more intensification of the amorphous peak as increasing the azido concentration, which was further supported by the narrower free volume distribution revealed by positron annihilation lifetime spectroscopy (PALS) analyses [57]. Likewise, our results imply that the free volume distribution in FDA2-MAT becomes narrower and thus potentially contributes to the improved selectivity of FDA2-MAT. At the same time, the large free volume provided by the bulky fluorinated diamine increases its gas permeability [1,58], consistent with the unique gas separation properties of FDA2-MAT. However, the T_g_ of FDA2-MAT is higher than that of FDA1-MAT, which is counterintuitive to the above explanation that the high free volume in FDA2-MAT is responsible for the improved chain mobility and rearrangements (Figure 7d). This contradiction may be ascribed to the lowest reactivity of bulkier FDA2 diamine, which is likely to be confined to the membrane surface compared with other diamine modifiers [27,31]. In this sense, more detailed characterization is recommended for future studies on diamine-modified polyimide membranes, particularly based on surface-sensitive analytical tools such as depth profiling, which is beyond the scope of the present work.

## 4. Conclusions

We explored the effects of fluorinated diamines in modified Matrimid^®^ 5218 membranes, which is a representative commercial polyimide. FT-IR, XPS, TGA, and gel fraction analyses confirmed the successful incorporation of both non-fluorinated and fluorinated diamines. The reactivity of a diamine depends on its molecular size, and thus the reaction between diamines and polyimide backbone may be constrained at the membrane surface due to the limited diffusion of diamine molecules. Gas transport properties of modified Matrimid membranes relied more on the kinetic diameter of the gas molecules compared to the pristine membrane, which indicates the improved diffusivity selectivity due to the crosslinked structure that contributes to the more efficient separation of gas pairs with larger size differences (e.g., H_2_/CH_4_ and CO_2_/CH_4_). Most importantly, the Matrimid membranes modified with fluorinated diamines displayed a simultaneous increase in both permeability and selectivity, overcoming the prevalent trade-off relationship that most polymer membranes suffer from. Detailed structural characterization results suggest that the bulky fluorine group is responsible for the expansion of free volume elements as well as chain rearrangement, which results in a more permeable membrane compared to the pristine one. Moreover, the partial crosslinking of Matrimid by forming amide bonds account for the improved selectivity of modified membranes. Overall, the diamine modification based on the fluorine-containing modifiers may offer an effective strategy to tailor the gas separation performance of polyimide membranes. Future work will be focused on the application of the same strategy to other polyimides and the fabrication of their thin-film composite (TFC) membranes for practical applications.

## Figures and Tables

**Table 1:** XPS elemental analysis of fluorinated diamines-modified Matrimid membranes.

Membranes	Atomic Concentration (at.%)			
	C	N	O	F
Mat	81.44	4.13	14.43	-
FDA1-MAT	77.83	4.11	13.95	4.11
FDA2-MAT	81.08	4.09	12.35	2.48

**Table 2:** Gas separation performance of diamine-modified Matrimid membranes at 2 bar and 35 °C.

Gas	Gas Permeability (Barrer)					
	MAT	DA0-MAT	DA1-MAT	DA2-MAT	FDA1-MAT	FDA2-MAT
H_2_	21.6	13.9	23.1	23.3	47.0	41.0
CO_2_	8.3	2.8	7.1	6.3	14.0	16.7
O_2_	1.9	0.9	1.4	1.6	3.1	3.4
N_2_	0.3	0.09	0.2	0.2	0.5	0.5
CH_4_	0.3	0.07	0.1	0.1	0.4	0.3
**Gas pair**	**Selectivity (-)**					
H_2_/N_2_	72	152	105	117	98	77
H_2_/CH_4_	79	186	199	166	119	114
O_2_/N_2_	6.3	9.3	6.5	7.9	6.5	6.5
CO_2_/N_2_	28	31	32	31	29	32
CO_2_/CH_4_	31	38	61	45	35	46

**Table 3:** Comparison of CO_2_/CH_4_ separation performance of amino-modified polyimide membranes.

Polyimide	Modifier	CO_2_ Permeability (Barrer)	CO_2_/CH_4_ Selectivity (-)	CO_2_ Permeability Enhancement (%)	CO_2_/CH_4_ Selectivity Enhancement (%)	Ref.
Matrimid	*p*-XDA	1.9	28	−71	−18	[26]
Matrimid	EDA	1.2	27.7	−81	−19	[31]
Matrimid	BuDA	2.1	18.7	−68	−45	[31]
Matrimid	*p*-PDA	4.2	46.1	−58	+15	[38]
Matrimid	*p*-PDA	9.5	45.9	−6	+14	[39]
6FDA-durene	DABD	244.8	23.6	−60	+74	[28]
6FDA-durene	CHBA	55.2	19.3	−91	+42	[29]
6FDA-durene	EDA	21.2	22.3	−97	+64	[30]
6FDA-durene	EDA	435	21.1	−29	+55	[31]
6FDA-durene	PDA	81.1	27.8	−87	+104	[31]
6FDA-durene	BuDA	218	24.7	−64	+82	[31]
6FDA-ODA	*p*-XDA	10.6	58.8	−76	+97	[47]
6FDA-ODA	n-ethylamine	7.8	37.5	−82	+25	[47]
6FDA-ODA	n-butylamine	8.8	42.9	−80	+43	[47]
Matrimid	DA1	7.1	61	−14	+97	This work
Matrimid	DA2	6.3	45	−24	+45	
Matrimid	FDA1	14	35	+69	+13	
Matrimid	FDA2	16.7	46	+101	+48	

Note: *p*-XDA = *p*-xylylenediamine, EDA = ethylenediamine, PDA = 1,3-propanediamine, BuDA = 1,4-butanediamine, *p*-pDA = *p*-phenylenediamine, DABD = diaminobutane dendrimer, CHBA = 1,3-cyclohexanebis(methylamine).

**Table 4:** The density of diamine-modified Matrimid membranes.

Membranes	Density (g/cm^3^)
MAT	1.242 ± 0.008
DA0-MAT	1.265 ± 0.009
DA1-MAT	1.248 ± 0.012
DA2-MAT	1.252 ± 0.009
FDA1-MAT	1.245 ± 0.009
FDA2-MAT	1.240 ± 0.008

## Data Availability

Not applicable.
